# Repetitive Grooming Behavior Following Aversive Stimulus Coincides with a Decrease in Anterior Hypothalamic Area Activity

**DOI:** 10.1523/ENEURO.0417-24.2024

**Published:** 2025-01-28

**Authors:** Brenton T. Laing, Megan S. Anderson, Aishwarya Jayan, Anika S. Park, Lydia J. Erbaugh, Oscar Solis, Danielle J. Wilson, Michael Michaelides, Yeka Aponte

**Affiliations:** ^1^Neuronal Circuits and Behavior Section, National Institute on Drug Abuse Intramural Research Program, National Institutes of Health, Baltimore, Maryland 21224-6823; ^2^Department of BioMolecular Sciences, School of Pharmacy, University of Mississippi, Oxford, Mississippi 38677; ^3^Biobehavioral Imaging and Molecular Neuropsychopharmacology Section, National Institute on Drug Abuse Intramural Research Program, National Institutes of Health, Baltimore, Maryland 21224-6823; ^4^Department of Psychiatry and Behavioral Sciences, Johns Hopkins University School of Medicine, Baltimore, Maryland 21205; ^5^The Solomon H. Snyder Department of Neuroscience, Johns Hopkins University School of Medicine, Baltimore, Maryland 21205

**Keywords:** anterior hypothalamic area, functional imaging, optogenetics, repetitive grooming behavior, ventromedial hypothalamus

## Abstract

The anterior hypothalamic area (AHA) is a key brain region for orchestrating defensive behaviors. Using in vivo calcium imaging in mice, we observed that AHA neuronal activity increases during footshock delivery and footshock-associated auditory cues. We found that following shock-induced increases in AHA activity, a decrease in activity coincides with the onset of grooming behavior. Next, we optogenetically activated the projections from the ventromedial hypothalamus (VMH) to the AHA and observed that photoactivation of the VMH→AHA pathway drives avoidance. Interestingly, repetitive grooming behavior occurs following cessation of stimulation. To identify changes in brain-wide activity patterns that occur due to optogenetic VMH→AHA stimulation, we combined optogenetic stimulation with positron emission tomography (PET)-based metabolic mapping. This approach revealed the amygdala as a downstream area activated by the stimulation of this pathway. Our findings show that the rise and fall of AHA neuronal activity triggers repetitive grooming behavior following learned fear and optogenetic stimulation. In addition, activation of the VMH→AHA pathway triggers changes in the activity patterns of downstream brain regions that are reported to be associated with displacement grooming.

## Significance Statement

This work identifies an association between grooming behavior and anterior hypothalamic area (AHA) activity patterns. Regardless of whether the activation of the AHA is initiated by shock-associated conditioned fear or optogenetic stimulation of excitatory inputs from the ventromedial hypothalamus, repetitive self-grooming behavior emerges during the postactivation decrease in activity. Further, this work identifies that even in anesthetized mice, AHA activation serves as a trigger for downstream network changes in the amygdala and striatum, which are associated with repetitive behavior. Together, this work positions the anterior hypothalamic area as a potential etiological factor to be considered at the intersection of fear experience and subsequent repetitive behaviors.

## Introduction

Association of salient cues with threatening stimuli aids in prediction of environments that may present threats (Canteras et al., 2008; Holtz et al., 2012; Wright and McDannald, 2019). Previous studies have demonstrated that the ventromedial hypothalamus (VMH) and the anterior hypothalamic area (AHA) are critical to this process. Escape behaviors such as increases in locomotor activity and jumping are triggered by activation of the VMH→AHA pathway (Wang et al., 2015). While VMH output specifically encodes for innate threats (Tobias et al., 2023), hippocampal inputs to the AHA promote contextual control of defensive behaviors (Bang et al., 2022b). Thus, the AHA serves as a hub for the convergence of innate and conditioned threat information to coordinate escape responses.

While previous reports demonstrated that AHA circuitry is involved in defensive behaviors, little is known about the behavioral effects elicited by fluctuations in AHA activity patterns that are driven by threat. Previous studies showed that recurrent activation of other fear-associated brain circuits leads to persistent repetitive grooming behavior (Hannigan and Isaacson, 1981; Ahmari et al., 2013). Therefore, we sought to investigate whether a change in AHA and VMH→AHA neuronal activity is temporally related to the onset of grooming behavior after fear conditioning.

Here, we addressed this by using a combination of in vivo functional imaging, optogenetics, and behavioral assays. First, the genetically encoded calcium indicator GCaMP and a detachable miniscope were used to record AHA neuronal activity in freely moving mice (Akerboom et al., 2012) during a classic footshock-cue conditioning paradigm (Vazdarjanova and McGaugh, 1998). We analyzed AHA neuronal activity changes during footshock delivery, footshock-associated auditory cues, and the relationship of these activity patterns to grooming behavior. Second, we used optogenetics to activate the VMH→AHA pathway and measured behavioral outputs such as locomotor activity, number of jumps, and grooming. Finally, we sought to identify downstream brain regions that become activated during VMH→AHA photoactivation by combining whole brain metabolic mapping and optogenetics. We observed that the amygdala and ventral striatum are downstream areas activated by stimulation of the VMH→AHA pathway.

## Materials and Methods

### Animals

All experimental protocols were conducted in accordance with US National Institutes of Health Guidelines for the Care and Use of Laboratory Animals and with the approval of the National Institute on Drug Abuse Animal Care and Use Committee. Male and female wild-type (C57BL6/J background; RRID:IMSR_JAX:000664; The Jackson Laboratory), *Vgat^Cre^* (*Slc32a1^tm2(cre)Lowl^*; C57BL/6J background; RRID:IMSR_JAX:028862, The Jackson Laboratory), and *Vglut2^Cre^* (*Slc17a6^tm2(cre)Lowl^*; C57BL/6J background; RRID:IMSR_JAX:028863, The Jackson Laboratory) mice were used. Prior to surgery, mice were group housed with littermates in temperature- and humidity-controlled rooms on a 12 h light/dark cycle with *ad libitum* access to water and rodent chow (PicoLab Rodent Diet 20, 5053 tablet, LabDiet/Land O’Lakes). Groups were approximately age and sex matched prior to surgery.

### Stereotaxic viral injections

Microinjections were performed using a stereotaxic apparatus (David Kopf Instruments) and micromanipulator (Narishige International) with custom-pulled glass pipettes (20 − 30 µm tip inner diameter). Mice were anesthetized using isoflurane (4% for induction and 1.5–2% for surgery) and administered postoperative ketoprofen (5 mg/kg, s.c.) for analgesia. For functional imaging experiments, an adeno-associated virus (AAV) was injected into the anterior hypothalamic area (AHA; 4 × 50 nl for 200 nl total; AP: −0.70 and −0.94, ML: +0.425, DV: −5.50 and −5.30) for the expression of the genetically encoded calcium indicator GCaMP (Akerboom et al., 2012). After viral injection, a 25-gauge sterile syringe needle was lowered to the implant depth to create a path for GRIN lens implantation and to avoid tissue compression. Finally, a GRIN lens (500 µm diameter; Snap-in Imaging Cannula Model L-V, Doric Lenses) was implanted dorsal to the AHA in accordance with the 80 µm working distance of the microscope (AP: −0.85, ML: +0.45, DV: −5.22). For behavioral and PET scan experiments, viral injections into the VMH (AP: −1.7, ML: +0.3, DV: −5.75, 50 nl) were performed, and custom-fabricated optical fibers (Sparta et al., 2011; 200 µm core, 0.48 NA, 4.8 mm length) were unilaterally implanted dorsal to the AHA (AP: −0.85, ML: +0.45, DV: −4.8). For optogenetic experiments, no control mice were excluded. However, channelrhodopsin (ChR2)-expressing mice were excluded if predominant viral expression was not medial to the fornix and targeted at the ventral half of the third ventricle. We excluded *n* = 4 ChR2-expressing mice that did not meet this criterion. A total of *n* = 11 mice in the control group and *n* = 7 ChR2-expressing mice in the stimulation group were used for experiments.

Viruses used include the following: (1) rAAV9/SYN-jGCaMP7s-WPRE, titer, 5.0 × 10^12^ GC/ml (Addgene viral prep # 104487-AAV9, RRID:Addgene_104487; Dana et al., 2019); (2) rAAV1/Camk2a-hChR2(H134R)-EYFP-WPRE, titer, 5.0 × 10^12^ GC/ml (Addgene viral prep # 26969-AAV1, RRID:Addgene_26969; Addgene; Lee et al., 2010); (3) rAAV1/Camk2a-eYFP-WPRE, titer: 5.0 × 10^12^ GC/ml (Addgene viral prep # 105622-AAV1, RRID:Addgene_105622); (4) rAAV1/CAG-FLEX-tdTomato-WPRE, titer, 5.0 × 10^12^ GC/ml (Addgene viral prep # 28306-AAV1, RRID:Addgene_28306).

### Footshock conditioning during functional imaging

Functional imaging in freely moving mice was performed by recording jGCaMP7s fluorescence from AHA neurons with a Doric Lenses microendoscope interfaced with an implanted GRIN lens (Laing et al., 2021) using Doric Neuroscience Studio software v5.1 (RRID:SCR_018569). Approximately 4 weeks after surgery, mice were habituated to experimenter handling 5 min/day for 1 week. Experiments were conducted ∼5 weeks postsurgery. This included 1 d of footshock conditioning and 1 d of cue-response testing (Vazdarjanova and McGaugh, 1998). Each day consisted of a 5 min test. The protocol for Day 1 (i.e., 120 s wait, 10 s tone, 5 s footshock, 120 s wait, 10 s tone, 5 s footshock, 35 s extra recording) was like the protocol for Day 2 (i.e., 120 s wait, 10 s tone, 125 s wait, 10 s tone, 5 s, 35 s extra recording) except the footshock was omitted on Day 2. Acquisition parameters were set to 100 ms exposure and 0 gain. Illumination power remained constant across sessions. Videos were motion corrected (Pnevmatikakis and Giovannucci, 2017), and fluorescence signals were extracted and converted into normalized intensity values as *Z*-score using the CaImAn miniscope pipeline (Giovannucci et al., 2019). The microscope and behavior camera were synchronized with triggering from ANY-Maze TTL cables. Freezing behavior was identified using ANY-maze video tracking software v6 (RRID:SCR_014289; Stoelting) with default settings and 1 s detection time. Grooming behavior was manually analyzed and time stamped. Custom MATLAB scripts were used for peri-event alignment to protocol and behavior time stamps (MATLAB R2020a, RRID:SCR_001622; MathWorks). These scripts compile the time-aligned data from the calcium imaging and behavior analysis. The data were analyzed 3 s before the onset of grooming behavior and 3 s following the onset of grooming behavior. Grooming bouts that were shorter than 3 s were excluded from calcium imaging analysis to ensure the analysis period coincided with continuous grooming behavior. For each cell, the activity patterns were averaged across all grooming bouts and the average and error across cells are reported. For conditioned fear experiments, the triggering to synchronize the mouse behavior and miniscope recording was improperly set up so the data from that mouse were excluded from analysis that required behavior data and calcium imaging data (e.g., correlation between activity patterns and speed, relationship between activity patterns and grooming). Freezing behavior was identified by using ANY-maze video tracking software with default settings and a 1 s detection time.

### Optogenetic manipulations and behavioral assays

All behavioral tests were conducted within the light phase. Mice were acclimated to the testing room for at least 1 h. Before testing, mice were tethered to a 450 nm laser (Doric Lenses) via optical fiber patch cords. The photostimulation protocols (10 ms pulse width at 20 Hz) were generated using Doric Neuroscience Studio software v5.1. Optogenetic stimulation intensity was selected in accordance with the targeted distance of the optical fiber (300–400 µm) from the ventral portion of the AHA to ensure sufficient illumination throughout the AHA. At this distance the power is attenuated to ∼29 or 23% of the power at the tip, respectively, according to the Stanford Irradiance calculator based on measurements in mammalian brain tissue (https://web.stanford.edu/group/dlab/cgi-bin/graph/chart.php). Videos were manually scored for jumping, grooming, and rearing behaviors with ANY-maze software v6 by an observer blinded to treatment groups. Notably, grooming was defined as licking, rubbing, scratching, or nibbling at any part of the body. Time spent immobile is a combination of periods where the mouse is still (i.e., unmoving) along the *x–y* axis of the apparatus including during rearing behavior as well as grooming behavior.

### Open field test

To detect the effects triggered by photoactivation of the VMH→AHA pathway, mice were placed in open field arenas (30 × 30 cm) with bedding on the arena floor. Arenas were placed inside isolation chambers illuminated to ∼150 lux. The 18 min testing session was divided into six alternating 3 min epochs. This bin length was demonstrated to modulate amygdala-dependent behavior (Felix-Ortiz and Tye, 2014). During the first, third, and fifth epochs, the laser was OFF. During the second, fourth, and sixth epochs, the laser was ON. An overhead camera and ANY-maze were used to record and assess mouse locomotion and location.

### Real-time place preference

For real-time place preference (RTPP) experiments, a standard-sized rat cage (20 × 40 cm) with black opaque walls and a layer of bedding was placed in an isolation chamber with the overhead lights turned off. Laser stimulation (20 Hz, 10 − 15 mW) was paired with one side of the chamber (laser-ON side) and was consistent across sessions. Mice were placed in the laser-OFF side and could freely transition between the two sides for 20 min. Average speed and total time spent in each side of the chamber were calculated by ANY-maze. Immobility was defined as the absence of movement in the *x*, *y*, and *z* space (Wang et al., 2015). For immobility detection, the sensitivity slider was set to 80% with a minimum duration threshold of 2 s. Grooming was manually scored offline.

### Persistent stimulation

Long-term optogenetic stimulation can trigger changes different from those during short-term stimulation (Abe et al., 2019). To measure the effects of extending the photoactivation time course, mice were placed in a testing apparatus for 3 h/day over 3 consecutive days (Days 1–3). During sessions, mice were tethered to a laser and received light pulses (10 ms, 20 Hz, 10 − 15 mW) for 3 h. An additional 3 min of video was acquired each day after shutting off the lasers to obtain postsession grooming scores. Data analysis was performed in MATLAB (R2020a, RRID:SCR_001622; MathWorks) to extract animal velocity 5 s before, during, and after photostimulation.

### Positron emission tomography

Mice were fasted for ∼16 h before the experiment. On the day of the experiment, mice were anesthetized with 2% isoflurane and placed on a custom-made bed in a NanoScan small animal PET/computed tomography (CT) scanner (Mediso Medical Imaging Systems). Mice were then injected (i.p.) with 13 MBq of 2-deoxy-2-[18F]fluoro-d-glucose (FDG; Cardinal Health) and scanned for 30 min using a dynamic acquisition protocol followed by a CT scan. During FDG uptake, mice were photostimulated using 3 min OFF/ON blocks (10 − 15 mW, 10 ms pulse, 20 Hz). PET data were reconstructed and corrected for dead-time and radioactive decay (Thanos et al., 2013). All qualitative and quantitative assessments of PET images were performed using the PMOD software environment (RRID:SCR_016547; PMOD Technologies) and Mediso's Nucline software. Data were reconstructed in frames corresponding to the blocks of stimulation, and the dynamic PET images were coregistered to magnetic resonance imaging templates using PMOD's built-in atlases. All statistical parametric mapping analyses were performed using MATLAB R2016 and SPM12 (RRID:SCR_007037; https://www.fil.ion.ucl.ac.uk/spm/software/spm12/; University College London) and evaluated at the *p* < 0.05 level using the Probabilistic Threshold-Free Cluster-Enhancement (pTFCE) method and multiple-comparisons correction (Spisak et al., 2019) with a cluster extent correction threshold of 100 contiguous voxels (*k* = 100).

### Histology

Mice were anesthetized with isoflurane and transcardially perfused with 1× phosphate-buffered saline (PBS) followed by 4% paraformaldehyde (PFA) in 1× PBS. Brains were removed and postfixed in 4% PFA at 4°C for at least 24 h. Tissue was embedded in 4% agarose/PBS and 50 µm free-floating, coronal brain sections were collected using a vibratome (Leica VT1200S, RRID:SCR_020243; Leica Biosystems), mounted with DAPI Fluoromount-G aqueous mounting medium (Electron Microscopy Sciences) onto Superfrost Plus glass slides (VWR International), and imaged with an Axio Zoom.V16 fluorescence microscope (Carl Zeiss Microscopy) using Zen 2012 software (Carl Zeiss Microscopy).

For representative images of axon fields in the AHA, sections were immunostained for GFP using chicken anti-GFP polyclonal antibody (1:1,000, catalog #GFP-1020, RRID:AB_10000240, Aves Labs). Slices were washed in PBS six times for 10 min and then blocked for 1 h in PBS/0.3% Triton X-100/3% normal donkey serum. Samples were incubated overnight at room temperature in primary antibody diluted in block solution. Then, slices were washed (6 × 10 min in PBS) followed by incubation in secondary antibody in block solution: goat anti-chicken Alexa Fluor 488 (1:500, catalog #A11039, RRID:AB_2534096; Thermo Fisher Scientific). Slices were then mounted onto Superfrost Plus glass slides (VWR International) and imaged with an Axio Zoom.V16 fluorescence microscope using Zen 2012 software or Keyence BZ-X710 (Keyence).

### Experimental design and statistical analyses

Data are reported as mean ± SEM. GraphPad Prism 8 software (RRID:SCR_002798; GraphPad) was used for all graphs and statistical analyses. Statistical tests are reported in detail in Extended Data Table 1-1. Student's *t* test was used to determine differences between groups when there were not repeated measures. QQ plots and residual plots were assessed for normality. For Student's *t* tests, Welch's correction was applied when variance was different between groups. Two-way repeated-measures ANOVA was used to detect differences between groups and within groups when appropriate. Sphericity was not assumed and Greenhouse–Geiser corrections were made for all experiments. Sidak's multiple-comparisons tests were used for further evaluation when significant main effects were detected. For multiple comparisons of footshock testing experiments, only comparison against the pretest baseline was conducted. The number of experimental units is depicted as “*n*” for number of mice for each experiment, except for the functional imaging data where “*n*” is the number of neurons.

### Code accessibility

The code described in the paper is freely available online at Open Science Framework (https://doi.org/10.17605/OSF.IO/U5BTN). The code is available as Extended Data.

10.1523/ENEURO.0417-24.2024.d1 Extended Data Download Extended Data, ZIP file.

## Results

### Footshock conditioned auditory cues evoke an increase in AHA neuronal activity

**Figure 1.**
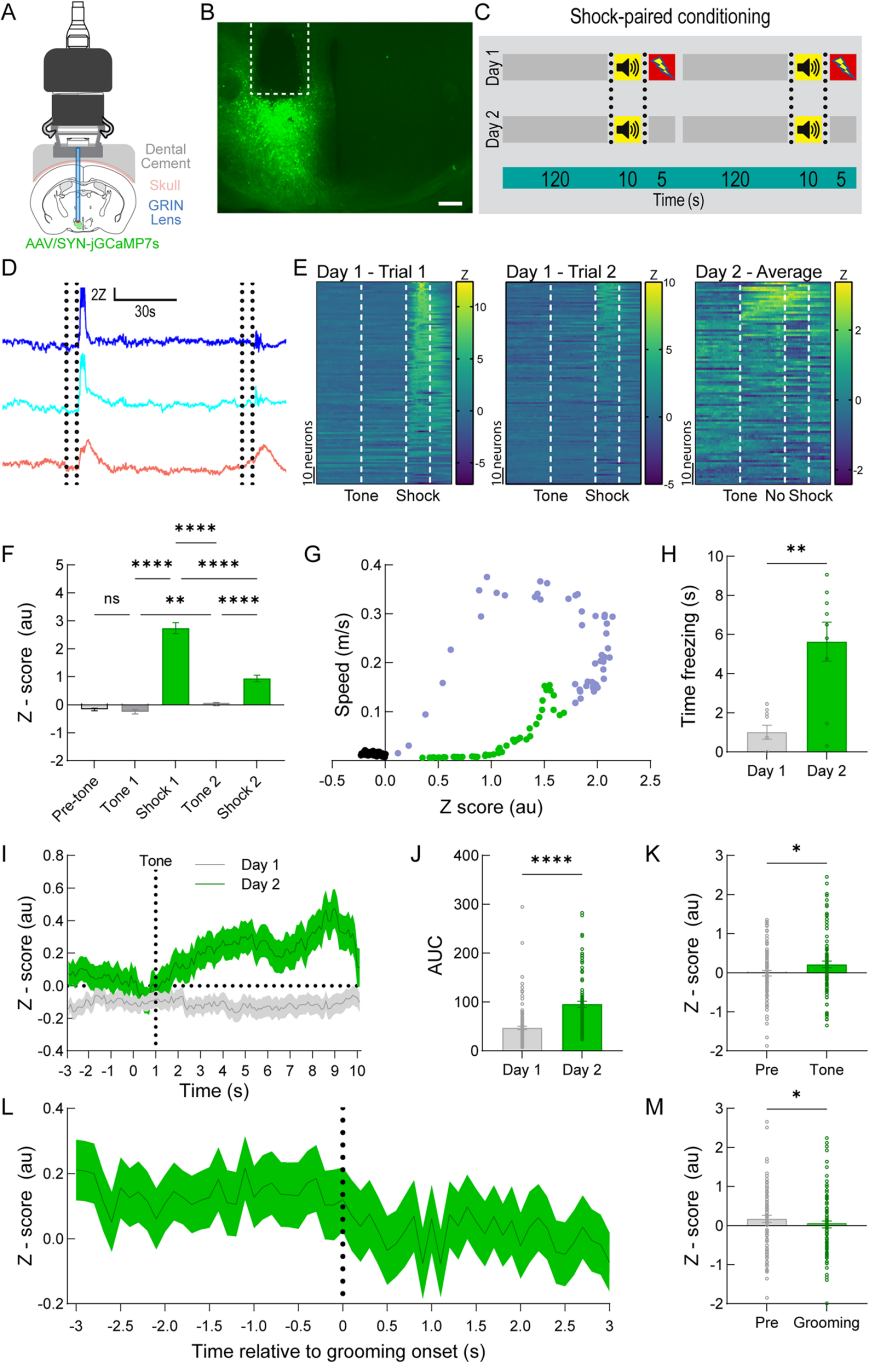
Changes in AHA neuronal activity during footshock-cue conditioning. ***A***, Strategy for expression of jGCaMP7s with GRIN lens implantation for imaging calcium-dependent signals in the AHA. ***B***, Representative image of GRIN lens placement (white dotted lines) and viral microinjection of the AHA. Scale bar, 200 µm. ***C***, Experimental timeline depicting the shock-paired conditioning session on Day 1 and testing session on Day 2 with two trials for each session. ***D***, Representative *Z*-scored traces from individual neurons during a Day 1 session. For each pair of black dotted lines, the first line indicates the time of tone onset, and the second line indicates the time of shock delivery. ***E***, Heat maps showing changes in AHA neuronal activity for each neuron on Day 1 as well as mean response to the tone on Day 2. The three dotted lines indicate the onset of the tone (line 1), shock onset (line 2), and shock offset (line 3). Note that no shock was delivered on Day 2. ***F***, Comparison of calcium sensor signal intensity during shock and tone deliveries on Day 1 showed significant increases (*n* = 121 neurons) during shock 1 and shock 2 delivery compared with the prior stage, a significantly higher response to tone 2 compared with tone 1, and a significantly lower intensity level during shock 2 compared with shock 1. No effects were detected during tone 1 presentation on Day 1. ***G***, There was a significant correlation on Day 1 between average *Z*-scored fluorescence intensity (*x*-axis) from jGCaMP7s in AHA neurons with locomotor speed (*y*-axis, *n* = 8 mice). Black dots indicate data before the shock, purple dots indicate data during the shock, and green dots indicate data in the 5 s following the shock. ***H***, Freezing analysis indicates significantly increased freezing on Day 2 compared with Day 1 (*n* = 9 mice). ***I***, Peri-event analysis showing *Z*-scored AHA activity 3 s before (“Pre”) and the 10 s duration of the tone (*n* = 90 neurons, data shown as mean ± 1 SEM). ***J***, Area under curve analysis (AUC) indicates significantly higher levels of *Z*-scored AHA activity during tone delivery on Day 2 compared with Day 1 (*n* = 90 neurons). ***K***, Significantly increased *Z*-scored mean intensity of AHA neuronal response was observed following tone delivery on Day 2 (*n* = 90 neurons). ***L***, Peri-event analysis centered around the onset of grooming at time point zero (*n* = 90 neurons, data shown as mean ± 1 SEM). ***M***, *Z*-scored quantification of intensity changes showed significantly decreased activity in AHA neurons (*n* = 90 cells) during bouts of grooming compared with the pregrooming period on Day 2. Statistical tests are detailed in Extended Data Table 1-1.

A recent study showed that contextual learning influences AHA neuronal activity in part through hippocampal inputs that enhance goal-directed escape behavior (Bang et al., 2022b). Therefore, we first investigated whether a classic footshock-cue conditioning paradigm modulates AHA neuronal activity. We specifically targeted an AAV expressing jGCaMP7s (Dana et al., 2019) to AHA neurons and visualized their activity using a miniscope attached to an implanted GRIN lens (Fig. 1*A*,*B*) during shock-paired conditioning (Fig. 1*C*). Changes in neuronal activity were aligned with the stimulus events (Fig. 1*D*,*E*). We calculated the mean *Z*-score during each of the stimuli and analyzed them using one-way ANOVA with multiple comparisons (Fig. 1*F*; *F*_(2.002,240.3)_ = 120.6, *p* < 0.0001). We observed a significant increase in AHA activity during the shocks compared with the stage that preceded them (Shock 1 vs Tone 1: *p* < 0.0001; Shock 2 vs Tone 2: *p* < 0.0001). Moreover, AHA neuronal activity was significantly higher during the second tone compared with the first tone (*p* = 0.0097), while the first tone did not result in a change in AHA activity compared with the time preceding the tone (*p* = 0.2411). Furthermore, we observed that the increase of activity during shock exposure was transient given that the level was significantly lower in the subsequent period (*p* < 0.0001). Additionally, we checked for sex differences and found that while there was a significantly higher *Z*-score intensity from the AHA of male mice compared with females (*n* = 27 male cells, *n* = 94 female cells, *t*_(28.49)_ = 4.019, *p* = 0.0004), there was no difference during the response to the tone on Day 2 (*n* = 13 male cells, *n* = 77 female cells, *t*_(88)_ = 0.2320, *p* = 0.8171). Peri-event analysis averaging the AHA neuronal activity and locomotor behavior around shock periods on Day 1 revealed a significant correlation between these parameters (Fig. 1*G*; *r* = 0.4446, *p* < 0.001). The cue-shock conditioning was sufficient to drive significant increases in freezing behavior during tone exposure on Day 2 compared with Day 1 (Fig. 1*H*; *t*_(9.92)_ = 4.368, *p* = 0.0014). We aligned the average *Z*-scored GCaMP signals across all cells with the tone delivery (3 s Pre, 10 s Tone delivery) for Day 1 and Day 2 (Fig. 1*I*). We then calculated the average area under curve (AUC) for each cell in the 10 s tone period relative to the baseline of that cell in the 3 s period before the tone and found a significantly higher AUC on Day 2 compared with Day 1 using Welch's *t* test (Fig. 1*J*; *t*_(145)_ = 6.543, *p* < 0.0001). On Day 2 after shock-paired conditioning, the tone itself triggered a significant increase in AHA neuronal activity compared with the pre-tone period (Fig. 1*K*; *t*_(89)_ = 1.998, *p* = 0.0488) indicating that AHA neuronal activity is modulated by learned fear. Interestingly, we observed the emergence of grooming behavior during the shock-pair conditioning paradigm, and thus, we sought to examine the correlation between the behavior and AHA neuronal activity. Notably, grooming behavior is considered a shock-induced displacement behavior (Mu et al., 2020). We conducted peri-event analysis at the onset of grooming behavior on Day 2 by aligning the onset of grooming behavior with the *Z*-scored GCaMP signal (Fig. 1*L*). We detected a significant decrease in AHA activity corresponding to the transition from nongrooming to grooming periods (Fig. 1*M*; *t*_(82)_ = 2.423, *p* = 0.0176). Together, these results demonstrate (1) an increase and subsequent decrease in AHA neuronal activity during learned threat exposure, (2) an increase in AHA activity during shock-induced locomotion, and (3) a decrease in AHA activity corresponding to the onset of grooming behavior.

10.1523/ENEURO.0417-24.2024.t1-1 Table 1-1 Statistical analyses. Download Table 1-1, DOCX file.

### Cessation of photoactivation of the VMH→AHA pathway is followed by repetitive grooming behavior

**Figure 2.**
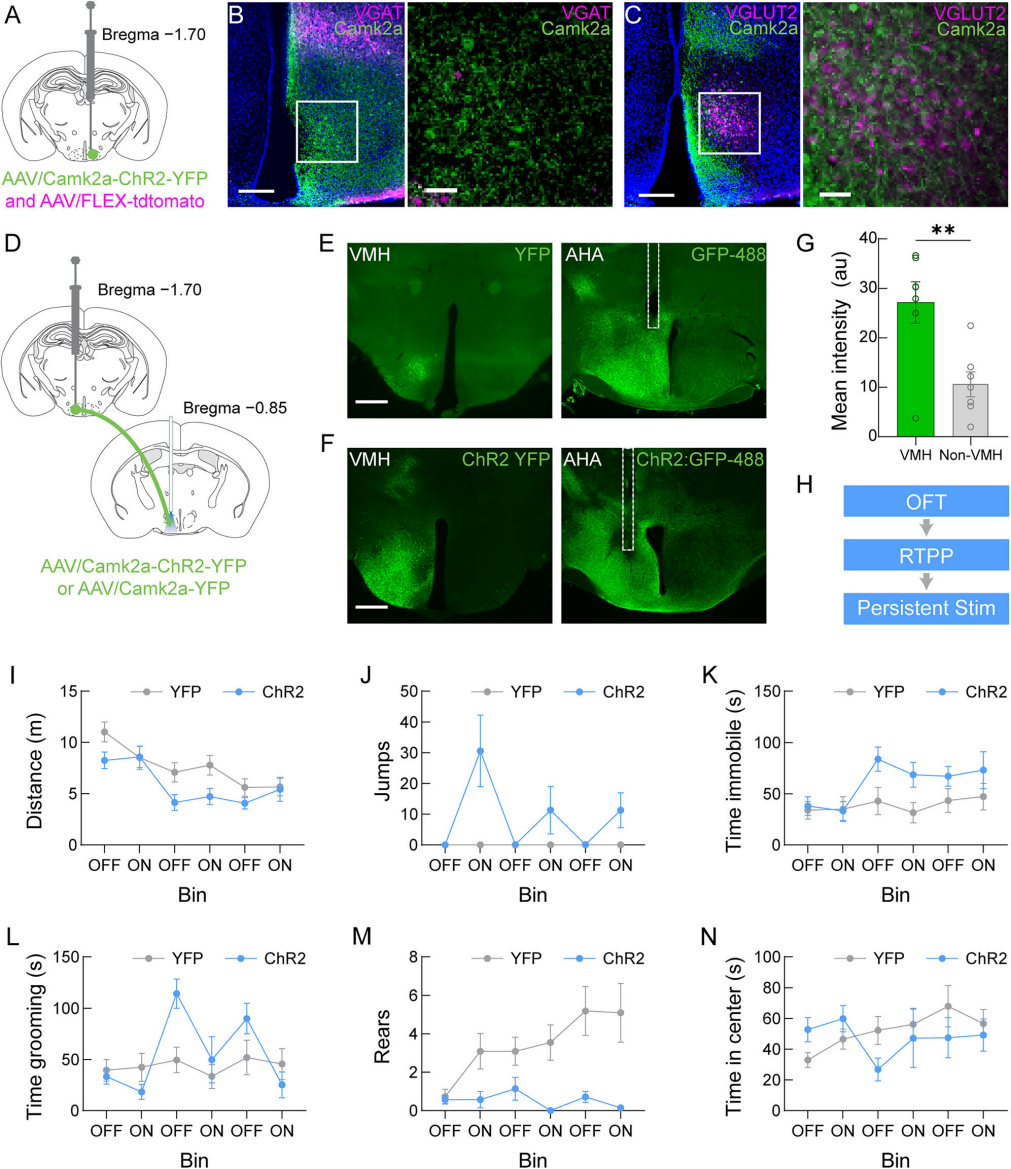
Photoactivation of the VMH→AHA pathway increases jumping and grooming behaviors. ***A***, Viral injection strategy to determine selectivity of AAV/Camk2a-ChR2-YFP for glutamatergic neurons. ***B***, Expression of AAV/Camk2a-ChR2-YFP does not colocalize with AAV/FLEX-tdTomato in the VMH of *Vgat^Cre^* mice. Scale bars: 200 µm (left), 50 µm (right). White box represents approximate area of zoomed image of the dorsomedial VMH (right). ***C***, Colocalization of AAV/Camk2a-ChR2-YFP and AAV/FLEX-tdTomato in the VMH of *Vglut2^Cre^* mice. Scale bars: 200 µm (left), 50 µm (right). White box represents approximate area of zoomed image of the dorsomedial VMH (right). ***D***, Viral microinjection strategy for the expression of AAV/Camk2a-ChR2-YFP or YFP control fluorophore in VMH neurons with an optical fiber implanted above the VMH axonal projections in the AHA. ChR2-expressing mice, *n* = 7; fluorophore control mice, *n* = 11. ***E***, Representative image depicting YFP fluorophore control expression in the VMH (left) and the immunostained (GFP-488) axon field with optical fiber tract (dotted white outline) in the AHA (right). Scale bar, 500 μm. ***F***, Representative image depicting ChR2-YFP expression in the VMH (left) and the immunostained (GFP-488) axon field with optical fiber tract (dotted white outline) in the AHA (right). Scale bar, 500 μm. ***G***, There was significantly greater fluorescence intensity in the VMH compared with other hypothalamic regions. ***H***, Diagram depicting order of optogenetic behavioral experiments. ***I***, No significant stimulation ×  transgene effect was detected for locomotor behavior in the OFT. ***J***, A significant stimulation ×  transgene interaction was detected for jumping behavior marked by higher levels of jumping in ChR2-expressing mice. ***K***, No stimulation ×  transgene effects were detected for time spent immobile. ***L***, A significant stimulation × transgene effect was observed for time spent grooming marked by a higher amount of grooming in ChR2-expressing mice compared with fluorophore controls. ***M***, A significant stimulation x transgene effect was observed for rearing behavior marked by reduced rearing in ChR2-expressing mice compared with fluorophore controls. ***N***, No effects on time spent in the center zone were detected. Statistical tests are detailed in Extended Data Table 1-1.

Previous studies showed that activation of the VMH→AHA pathway promotes escape behaviors (Wang et al., 2015). However, it is unclear whether photoactivation of this pathway could trigger grooming behavior. While others have conducted elegant work using the *Vglut2^Cre^* mouse model to target the VMH (Pinciotti et al., 2022), we sought to target the excitatory outputs from the VMH to the AHA with *Camk2a* promoter-driven viral vectors (Fig. 2*A*). First, we validated that the *Camk2a* promoter selectively expresses transgene in excitatory neurons by coinjecting an AAV/Camk2a-ChR2-YFP with AAV/FLEX-tdTomato in the VMH of *Vgat^Cre^* mice (Fig. 2*B*) or *Vglut2^Cre^* (Fig. 2*C*). We observed an absence of VMH^VGAT^ tdTomato-positive neurons but an abundance of VMH^VGLUT2^ tdTomato-labeled neurons in the dorsomedial VMH. AAV/Camk2a-ChR2-YFP expression was abundant in both mouse lines and only labeled VMH^VGLUT2^ neurons. Moreover, cellular compartmentalization of the tdTomato fluorophore (cytosolic) and channelrhodopsin-2 (ChR2; light-sensitive neuronal activator; membrane expressed) were noteworthy. Next, wild-type mice were unilaterally injected with AAV/Camk2a-ChR2-YFP or a control fluorophore into the VMH with an optical fiber implanted dorsal to the AHA (Fig. 2*D*). YFP control fluorophore (Fig. 2*E*) or ChR2-YFP (Fig. 2*F*) were visualized by postmortem histological analysis. Furthermore, ChR2-YFP expression in the VMH was quantified and was significantly higher than expression in regions adjacent to the VMH **(**Fig. 2*G*; *t*_(6)_ = 5.081, *p* = 0.0023). Notably, visualization of YFP and ChR2-YFP appear to have different cellular compartmentalization whereby YFP control fluorophore can be seen in the soma while the ChR2-YFP appears diffuse along axon fields. ChR2-YFP expression in the axon contributes to quantification of intensity outside of the VMH. These mice were utilized for subsequent behavioral experiments. For optogenetic manipulations during behavior, we first placed the mice in an open field apparatus (Gould et al., 2009) and photoactivated the VMH→AHA pathway using a fixed interval schedule for the delivery of light pulses (i.e., 3 min alternating periods between “ON” and “OFF”). We used two-way repeated-measures ANOVA to analyze the behavioral responses, and no significant differences were observed in locomotor distance (Fig. 2*I*; stim × transgene: *F*_(5,70)_ = 1.907, *p* = 0.1041), but significant increases were detected during jumping behavior (Fig. 2*J*; stim × transgene: *F*_(5,80)_ = 8.526, *p* < 0.0001). Moreover, we found that time spent immobile was not significantly different between groups (Fig. 2*K*; stim × transgene: *F*_(1,16)_ = 4.792, *p* = 0.1415). Furthermore, we manually scored rearing and grooming behaviors and found significant increases in the time spent grooming during the OFF epochs that followed photostimulation (Fig. 2*L*; stim × transgene: *F*_(5,80)_ = 5.027, *p* = 0.0005) and a significant suppression of rearing behavior (Fig. 2*M*; stim × transgene: *F*_(5,80)_ = 2.648, *p* = 0.0288) that persisted between photostimulation epochs. In addition, there were no significant effects of photostimulation on time in the center zone (Fig. 2*N*; stim × transgene: *F*_(5,70)_ = 2.409, *p* = 0.0449). Together, these results are consistent with our functional imaging recordings indicating that the onset of grooming behavior occurs immediately after the cessation of photoactivation of the VMH→AHA pathway.

**Figure 3.**
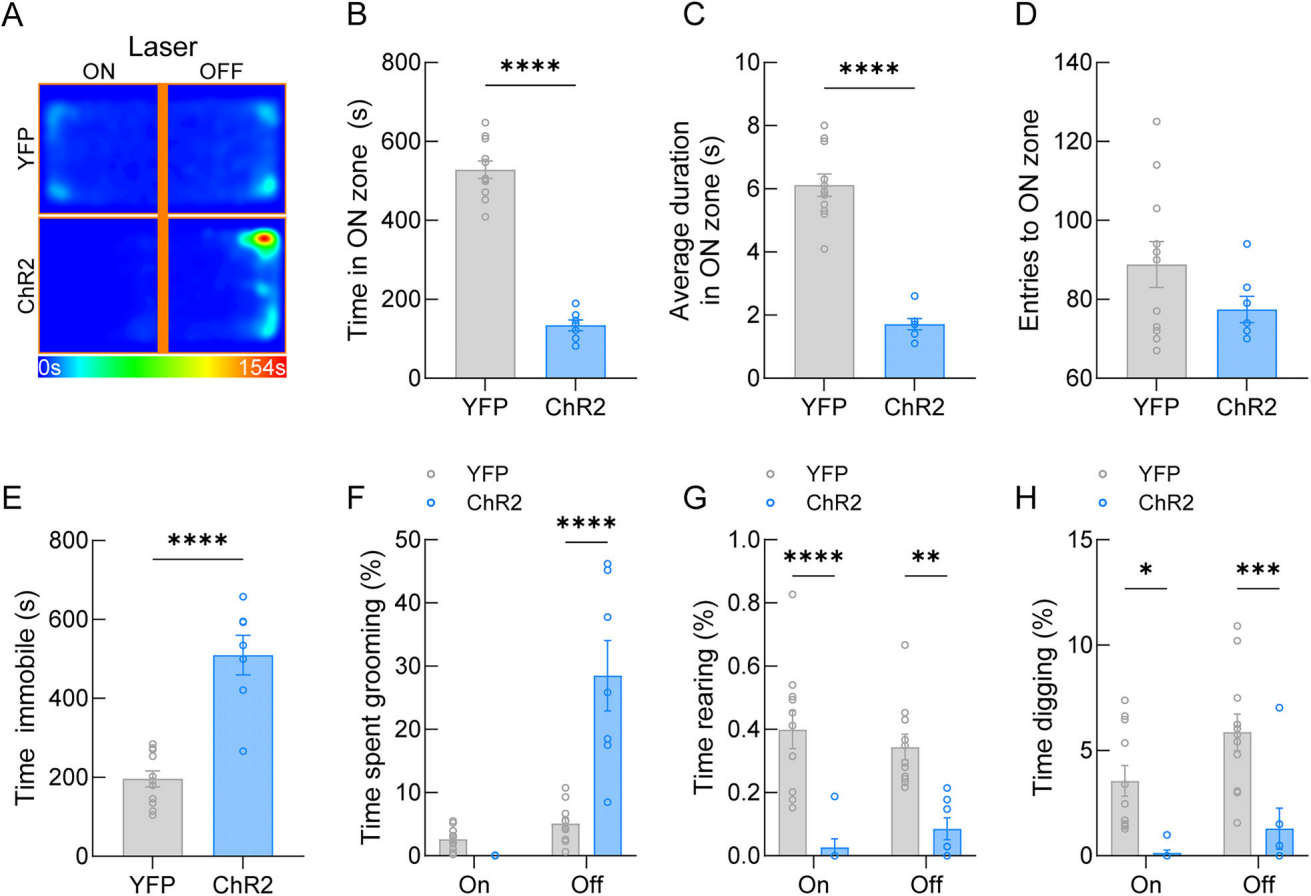
Photoactivation of the VMH→AHA pathway drives avoidance and OFF zone repetitive grooming behavior. ***A***, Mean heat maps for groups during RTPP showed that ChR2 photoactivation causes mice to spend more time in the Laser-OFF zone. ***B***, Time spent in the laser-paired ON zone was significantly reduced in ChR2-expressing mice compared with fluorophore controls. ***C***, The average duration in the Laser-ON zone was significantly reduced in ChR2-expressing mice compared with fluorophore controls. ***D***, There were no significant differences detected in the number of Laser-ON zone entries between groups. ***E***, Time immobile was significantly increased in ChR2 compared with fluorophore controls. ***F***, Time spent grooming in the Laser-OFF zone showed ChR2-expressing mice exhibit significantly more grooming behavior than fluorophore controls, even when normalized for time in the OFF zone. ***G***, A significant suppression of rearing behavior was observed in the ChR2-expressing mice compared with fluorophore controls in the Laser-ON and Laser-OFF zone. ***H***, VMH→AHA photostimulation resulted in a significant suppression of digging behavior in the Laser-ON and Laser-OFF zone. Statistical tests are detailed in Extended Data Table 1-1.

We next used RTPP (Bimpisidis et al., 2020) to determine the effects of VMH→AHA photostimulation on place preference or aversion. For this test, photostimulation was paired with one side of the arena (Laser-ON zone) and the laser remained off on the other side (Laser-OFF zone). Mice freely moved around the arena during testing, and heat maps were generated to detect mouse location (Fig. 3*A*). In congruence with previous indications that activation of the VMH→AHA pathway triggers avoidance behavior (Wang et al., 2015), we observed a significant decrease in the amount of time the ChR2-expressing mice spent in the Laser-ON zone of the arena compared with YFP control mice (Fig. 3*B*; *t*_(16)_ = 13.12, *p* < 0.0001). This was further indicated by a significant decrease in the average duration in the Laser-ON zone (Fig. 3*C*; *t*_(16)_ = 9.387, *p* < 0.0001). However, no significant changes were detected in the number of entries to the Laser-ON zone (Fig. 3*D*; *t*_(16)_ = 1.464, *p* < 0.1627) suggesting a potential lack of behavioral learning to avoid the aversive side of the arena. Additionally, ChR2-expressing mice spent significantly more time immobile during photoactivation (Fig. 3*E*; *t*_(16)_ = 6.735, *p* < 0.0001). For self-grooming behavior, we detected a significant effect of stimulation (Fig. 3*F*; *F*_(1,16)_ = 45.17, *p* < 0.0001), transgene (Fig. 3*F*; *F*_(1,16)_ = 21.06, *p* < 0.0003), and the interaction of these factors (Fig. 3*F*; *F*_(1,16)_ = 31.98, *p* < 0.0001). Sidak's multiple comparisons test detected a significant between groups difference specifically in the Laser-OFF zone (adjusted *p* < 0.0001) but not the Laser-ON zone (adjusted *p* = 0.6619). Significant transgene effects were detected for rearing (Fig. 3*G*; *F*_(1,16)_ = 26.17, *p* = 0.0001), but no stimulation (Fig. 3*G*; *F*_(1,16)_ = 0.0037, *p* = 0.9524) or interaction effects (Fig. 3*G*; *F*_(1,16)_ = 3.495, *p* = 0.0800) were detected. Sidak's multiple comparisons detected significant suppression of rearing behavior in both the Laser-ON zone (adjusted *p* = 0.0001) and Laser-OFF zone (adjusted *p* = 0.0014). Moreover, significant stimulation (Fig. 3*H*; *F*_(1,16)_ = 7.138, *p* = 0.0167) and transgene (Fig. 3*H*; *F*_(1,16)_ = 17.43, *p* = 0.0007) effects were detected for digging behavior. While no stimulation × transgene interaction effect was detected for digging behavior (Fig. 3*H*; *F*_(1,16)_ = 0.8011, *p* = 0.3840), Sidak's multiple comparisons detected significant suppression of digging behavior in both the Laser-ON zone (adjusted *p* = 0.0116) and Laser-OFF zone (adjusted *p* = 0.0008). Together, activation of the VMH→AHA pathway drives avoidance-like behaviors, and cessation of stimulation is associated with an increase in grooming behavior.

### Repeated intermittent VMH→AHA photoactivation evokes sustained grooming behavior

**Figure 4.**
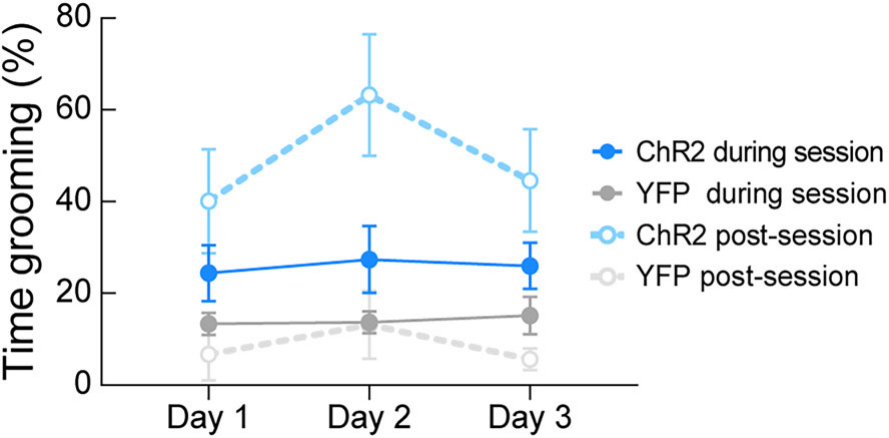
Repeated intermittent photoactivation of the VMH→AHA pathway promotes lasting grooming behavior. VMH→AHA stimulation increased the percentage of time spent grooming postsession compared with fluorophore controls (*p* < 0.05 each day). Multiple comparisons reveal that grooming is greater in ChR2-expressing mice on Day 2 after the session (post) compared with behavior during the session. Statistical tests are detailed in Extended Data Table 1-1.

The emergence of grooming behavior after exposure to predatory threat (Dell'Omo and Alleva, 1994) or environmental stress (Moyaho and Valencia, 2002) has been shown previously (Ahmari et al., 2013). Thus, we sought to determine the effects of repeated activation of the VMH→AHA pathway on grooming behavior. For this, we used a fixed interval schedule for the delivery of light pulses consisting of 5 s “ON” and 25 s “OFF” for 3 h on 3 consecutive days. Three-way repeated-measures ANOVA resulted in detection of a significant increase in grooming behavior, particularly in the poststimulation phase of the experiment (Fig. 4; transgene × phase: *F*_(1,82)_ = 14.59, *p* = 0.0003). All potential multiple comparisons were calculated with Holm–Sidak correction. Multiple comparisons revealed between-group differences in the poststimulation period each day (Day 1: *p* = 0.0110, Day 2: *p* < 0.0001, Day 3: *p* = 0.0060). Within-group comparisons did not reveal significant differences. These results demonstrate that prolonged grooming behavior is triggered by continuous photostimulation of the VMH→AHA pathway and is particularly significant and detectable for minutes after activation has ceased.

### Photoactivation of the VMH→AHA pathway increases neuronal activity in the amygdala

**Figure 5.**
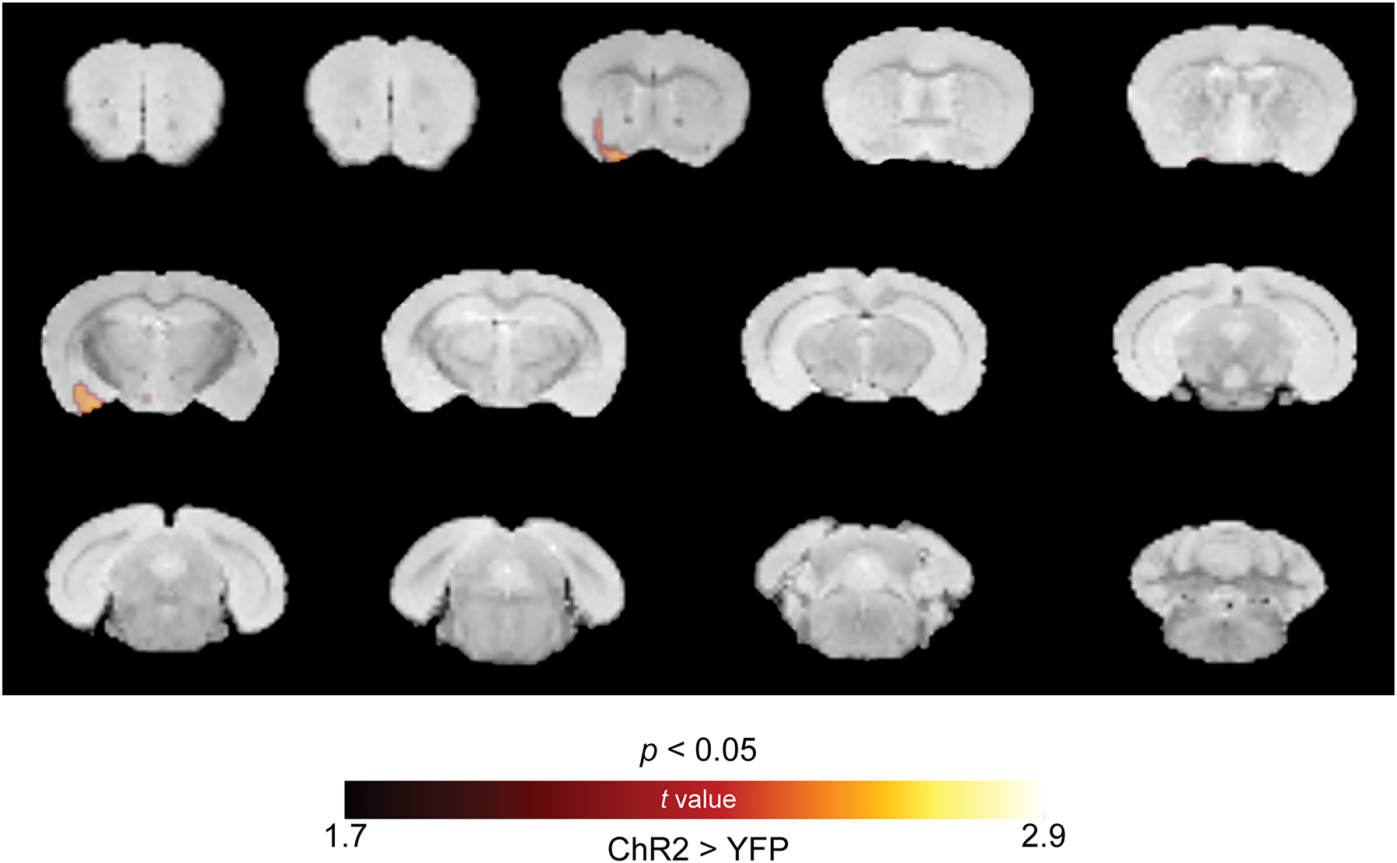
Photoactivation of the VMH→AHA pathway increases neuronal activity in the amygdala and ventral striatum. FDG-PET overlaid on structural MRI shows that VMH→AHA stimulation significantly increases FDG uptake compared with controls (*p* < 0.05, unpaired *t* test; *n* = 6 ChR2 and 10 YFP) in a region encompassing the lateral/ventrolateral striatum (left) and amygdala (right). Statistical tests are detailed in Extended Data Table 1-1.

We conducted whole brain metabolic mapping using 2-deoxy-2-[18F]fluoro-d-glucose (FDG)-PET while photostimulating the VMH→AHA pathway in anesthetized mice. For this, we compared the effects of VMH→AHA activation in ChR2-expressing mice and control mice. We found that photostimulation of the VMH→AHA pathway significantly increased FDG uptake (i.e., increased metabolic activity) in brain areas encompassing the lateral ventrolateral striatum and the amygdala (Fig. 5), which are brain regions known to be involved in the regulation of fear-related behaviors (Adolphs et al., 1995; Fanselow and Gale, 2003; Ehrlich et al., 2009). Notably, VMH→AHA pathway activation was not associated with any decreases in brain metabolic activity.

## Discussion

Hypothalamic circuitry is involved in controlling context-dependent defensive behaviors, particularly escape behaviors. Previous studies suggest that the VMH manipulates emotional state (Kunwar et al., 2015). The AHA regulates threat-induced behaviors (Wang et al., 2021) and corticosterone endocrine responses (Bang et al., 2022a). The AHA exerts effects across physiological systems controlling thermoregulation (Feldberg and Myers, 1965) and blood pressure (Hilton and Spyer, 1971) that facilitate selection within the repertoire of fight-or-flight behaviors (Rojas et al., 2012). While GABAergic neurons in the AHA are reported to promote biting behavior (Xie et al., 2022), glutamatergic parvalbumin neurons in the anterior hypothalamic area promote threat detection and defensive flight behavior (Laing et al., 2023). Further suggesting a role in selection of behavior from a complex defensive repertoire, electrolytic lesions of the VMH and AHA attenuate freezing in response to a predator odor (Pagani and Rosen, 2009). These studies strongly support a role of the AHA in detection and responding to environmental threats.

Here, we report that fear conditioning evokes an increase in AHA neuronal activity, and in the period that follows, a decrease in AHA activity is aligned with the onset of grooming behavior. Fiber photometry studies support that the VMH itself retains innate but not conditioned fear exposure (Tobias et al., 2023) while the hippocampus relays learned information (Bang et al., 2022b). These seminal studies demonstrate that the AHA serves as a complex hub that integrates multiple streams of information. Our study further explains the neurobiological processes that transition acute experiences into persistent repetitive behaviors.

We analyzed neuronal activity during the transition from nongrooming to grooming states and found a significant decrease in AHA neuronal activity at the onset of grooming. This striking observation indicates that a decrease in AHA activity corresponds to the onset of grooming behavior. One criticism for the measurement of neuronal activity changes using GCaMP is dependent parameter selection in which every analysis has some weakness (Jean-Richard-dit-Bressel et al., 2020). For feature-based analysis, mean values can be calculated from the acquired data which is effective for analysis of rewarding (Rossi et al., 2019) or fear conditioned auditory stimuli (Chen et al., 2023). We used mean *Z*-scored change in activity to overcome the weaknesses of using peak measures of frequency or amplitude. This approach is intuitive and brings the advantage of a single exact *p* value. In addition, we used jGCaMP7s to avoid any Type II errors attributable to event-related transient detection that is resilient to consecutive thresholds in proportion to the effect of activity change size. This analysis is corroborated with AUC calculations. While a key criticism of AUC analysis is the use of an arbitrary time window for calculations, our approach empirically overcomes a large portion of this issue by using the grooming events as the onset for the calculation. Further, we utilized an end-time for the window of 3 s, which is intuitive given the 3.75 s average grooming bout duration from the dataset. This 3 s window contained half of the total grooming bouts exhibited by all the mice throughout the sessions. Thus, our analysis uses a temporally defined window directly related to the behavior of the mice that overcomes the primary weakness of feature-based and AUC analysis. The rigor of these results is strengthened by multiple dependent variables for the assessment of our effect but also maintains the simple and intuitive benefits of common summary analysis methods. While these data do not causally implicate AHA activity in the control of grooming behavior, they clearly show that the changes in these parameters co-occur. Of note, the shock and conditioned tone responses were positive-going while the grooming onset response was negative-going. Thus, we did not compare fear and grooming responses directly.

We used optogenetics across three behavioral assays to elucidate the effects on behavioral outputs during and after photostimulation of the VMH→AHA pathway. First, we performed an open field test (OFT) as an unbiased assessment of behavior. We did not observe significant changes in locomotor activity, but our results in jumping behavior were consistent with previous findings (Wang et al., 2015) showing increases in jumping evoked by VMH→AHA activation. The discrepancy between the locomotor data is likely due to the longer duration of our time bins. While the illumination power at the tip of the fibers in the previous study was up to 6 mW and ours was 10–15 mW, their optical fiber core was 125 µm compared with the 200 µm core used in our study. Thus, it is likely that the irradiance at the target site was approximately equal between these studies. While unlikely to be a critical factor, another discrepancy was their use of a 20 ms stimulation duration while we used a 10 ms stimulation. Overall, the phenotype evoked by the stimulation reported here is consistent with this previous study.

A key advancement by our work is the demonstration of significant increases in grooming behavior between photostimulation sessions for the OFT. This observation following supraphysiological activation by optogenetic stimulation corroborates our observations using in vivo functional imaging that the onset of AHA activity attenuation coincides with grooming behavior initiation. Grooming is a displacement behavior that emerges during conflicts between behavioral systems (Spruijt et al., 1992). The persistent change in behavior that continues after fear exposure or optogenetic stimulation is in line with previous evidence of a persistent internal state following VMH activation patterns (Kennedy et al., 2020).

Next, we used the RTPP paradigm to identify behaviors evoked by VMH→AHA activation. We found that the average duration in the photostimulation-paired side of the arena was significantly reduced while no differences were observed for the total number of entries. These results suggest that either the mice were still driven to explore the photostimulation-ON zone or that AHA activity does not drive fear conditioning by itself. Nevertheless, the significantly reduced time in the laser-ON side of the apparatus indicates that VMH→AHA activation causes mice to avoid the stimulation. The emergence of grooming behavior during the RTPP test in the laser-OFF side is consistent with our OFT results that show increased grooming between stimulation bins. This indicates that a long duration of stimulation is not necessary to trigger this grooming behavior. We also saw significant reductions in typical mouse exploratory behaviors such as digging and rearing. Previous studies showed that elevation in grooming behavior occurs at the expense of behaviors related to exploration such as rearing and digging (Lever et al., 2006). Thus, our results are consistent with this previously identified phenomenon and indicate that the selection of grooming behavior has displaced those other behaviors. Our implementation of both the open field and RTPP tests led to the discovery of the emergence of grooming following stimulation offset if the stimulation was on the timescale of minutes or seconds. Notably, we observed that cessation of activation of the excitatory VMH→AHA pathway evokes an increase in grooming behavior.

Additionally, we examined the effects of persistent VMH→AHA photoactivation on grooming behavior using a fixed-interval stimulation paradigm. Remarkably, we found that fixed-interval stimulation significantly increased grooming behavior during the fixed-interval period and in the period following photostimulation sessions. Our results not only extend findings on the context-dependent roles that hypothalamic circuits play on escape vigor (Wang et al., 2021) but also demonstrate that persistent photostimulation of the VMH→AHA pathway evokes long-lasting grooming behavior. Changes in grooming behavior can be observed after rodents undergo stressful experiences (Kalueff and Tuohimaa, 2004), and self-grooming behavior relates to the degree of stress in an inverted-U curve relationship (Kalueff et al., 2016). In the absence of aversiveness, low self-grooming is observed, whereas exposure to stressful states increases grooming behavior and highly aversive experiences occlude grooming (Fernandez-Teruel and Estanislau, 2016). Importantly, in our experiments, grooming remained elevated following the 3 h session of persistent VMH→AHA stimulation. Our results indicate that the initial effect of stimulation may be adaptive to facilitate flight from an aversive stimulus and there is a subsequent shift in behavioral selection toward grooming. This is comparable with other studies that have found the emergence of grooming behavior after ChR2-mediated activation of escape (Mangieri et al., 2019). While our observations are consistent with those findings, it is not clear if the self-grooming behavior we observe is adaptive, maladaptive, or neutral. For example, a very elegant recent study showed an increase in self-grooming during stimulation of rewarding zona incerta neurons (Jiang et al., 2024).

The AHA projects to structures that are highly conserved within mammalian species, including the medial preoptic area, lateral hypothalamus, dorsomedial nucleus, capsule of the ventromedial nucleus, dorsal premammillary nuclei, and the central gray (Saper et al., 1978). Notably, the AHA consists predominantly of GABAergic neurons as well as a population of thyroid hormone-dependent parvalbumin neurons (Harder et al., 2018). The AHA receives projections from the lateral septum, paraventricular hypothalamus, ventromedial hypothalamus, paraventricular thalamus, and the ventral premammillary nucleus (Xie et al., 2022). Therefore, AHA circuitry integrates numerous synaptic inputs to modulate neuronal activity through downstream networks.

Using PET combined with optogenetic activation of the VMH→AHA pathway in anesthetized mice, we found that the lateral and ventrolateral regions of the striatum as well as the amygdala are downstream area activated by the stimulation of this pathway. The amygdala is a highly conserved structure involved in fear-related behaviors (Ohman, 2005). Previous studies showed that the central amygdala and bed nucleus of the stria terminalis serve as a subpallidal corridor that exhibits increased activity during exposure to aversive stimuli and uncertain/remote threat (Shackman and Fox, 2016). In mice, the activity of glutamatergic neurons in the amygdala increases in response to aversive stimuli (Cui et al., 2017). Moreover, self-grooming behavior is observed following stimulation of glutamatergic neurons in the amygdala (Jennings et al., 2013). Notably, we conducted comparative analysis between groups throughout the whole brain, and all the sections containing significant differences are shown in the figure. Together, our findings and those of others suggest that neurons within the hypothalamic→amygdala circuitry play a role in selection of behaviors that follow shock-induced threat. Remarkably, this circuitry is evoked even when the mouse has no perception of fear due to anesthesia. Notably, activity patterns in other brain regions may be influenced by VMH→AHA activity, but due to limitations in temporal and spatial resolution of the PET scan, these effects may not have been detectable.

In summary, our work revealed that the AHA responds to conditioned threat and that a decrease in conditioned or photoactivated AHA neuronal activity corresponds to the onset of repetitive grooming behavior. Moreover, we demonstrated that persistent intermittent photostimulation of the VMH→AHA pathway evokes sustained increases in grooming behavior. Furthermore, we showed that VMH→AHA photoactivation increases activity in the ventrolateral striatum and amygdala. Further investigations will be needed to examine VMH→AHA loss-of-function in ethologically relevant behavioral paradigms for context-dependent flexibility of behavioral selection. Our work highlights the need for future experiments to determine the specific neuron populations that modulate repetitive and defensive-related behaviors.

## Synthesis

Reviewing Editor: Sam Golden, The University of Washington Decisions are customarily a result of the Reviewing Editor and the peer reviewers coming together and discussing their recommendations until a consensus is reached. When revisions are invited, a fact-based synthesis statement explaining their decision and outlining what is needed to prepare a revision will be listed below. The following reviewer(s) agreed to reveal their identity: Shi-Bing Yang. Note: If this manuscript was transferred from JNeurosci and a decision was made to accept the manuscript without peer review, a brief statement to this effect will instead be what is listed below.

Dear Dr. Aponte,

Thank you for your patience as I have worked to receive reviewer comments without engaging fresh reviewers. Your manuscript, and the rebuttal from the JN submission, have been reviewed by one of the same experts, who was kind enough to also comment on the second set of reviews. I have also reviewed your manuscript to provide comments in relation to Reviewer 2's initial comments.

Overall, we are both enthusiastic for the eventual acceptance of this manuscript after some key issues have been addressed. Specifically, the reviewer appreciates the effort to address the concerns raised by the initial review. Reviewer 1 has some questions relating to the data quality and presentation. Reviewer 1 requested more detailed analytical data presentation. These comments can be resolved without full re-review, and the rebuttal will be editorially reviewed if submitted. Reviewer 2 has some questions relating to the data quality and presentation.

Below please find the specific comments.

Reviewer 1:

Based on the provided comments and rebuttal, it appears that the authors have made some efforts to address Reviewer 1's concerns, but several key issues remain only partially resolved:

1. AHA Activity and Grooming Behavior: The reviewer questioned whether the observed grooming behavior at stimulation offset is truly linked to AHA activity or could be a non-specific and non-physiological response associated with optogenetic stimulation. This raises doubt about the causal relationship between AHA activity and grooming. The authors referenced previous studies (e.g., Dayu Lin's work) to justify their stimulation parameters and adjusted their discussion to account for limitations in interpreting grooming behavior as causally linked to AHA activity. However, I think the link between AHA activity and grooming remains correlational. To fully address this concern, the authors need to provide stronger evidence or analyses that directly test whether AHA activity modulates grooming behavior, as requested by reviewer 1.

2. Single-Unit Calcium Imaging Analysis: Reviewer 1 suggested more in-depth analyses, such as waveform analysis or linear mixed models, which could yield insights into the neuronal mechanisms underlying the observed behaviors. The authors added event-related transient and AUC analyses to Figure 1 and displayed more cell-level distributions, aiming to align with typical calcium imaging standards. Nevertheless, the authors did not fully adopt the advanced analyses suggested by reviewer 1. This limits the depth of the analysis and the mechanistic insight into how neuronal activity correlates with behavior, which could have strengthened the study's findings.

3. Whole-Brain Imaging Data: The reviewer recommended a more detailed and comprehensive presentation of brain region activation patterns, including negative data to serve as controls. However, the authors clarified that only the significant activations are shown. I think Including these data could enhance understanding of the specific circuitry involved and provide a more rigorous control.

In conclusion, while the authors have made partial revisions, the current response does not fully address Reviewer 1's key recommendations for stronger causative evidence. Further revisions incorporating these elements would help clarify the findings and address the reviewer's concerns.

Reviewer 2:

After reviewing the rebuttal and revised manuscript, I appreciate the authors' efforts in addressing the concerns raised in the initial review. However, there are still areas where data quality and presentation can be strengthened, particularly regarding the authenticity of the data. Below are my specific comments:

To further strengthen the results, I encourage the authors to provide the raw data underlying their analyses. This will allow for a more thorough verification of the findings and help alleviate concerns about the reliability of some figures.

Several figures, notably Figure 1F and Figures 3F-H, appear to have been generated using graphical software rather than statistical software such as Prism. For example, the error bars in Figure 1F are not symmetric, and the outlines of the bars appear to protrude. These graphical inconsistencies raise questions about the figures' accuracy. I would suggest re-plotting these figures using appropriate statistical software to ensure consistency and accuracy in data presentation.

I recommend presenting a more detailed breakdown of the calcium imaging data, possibly including additional statistical analyses that highlight the variability across individual neurons rather than relying solely on mean Z-scores.
