# Supplementary material for: Repetitive Grooming Behavior Following Aversive Stimulus Coincides with a Decrease in Anterior Hypothalamic Area Activity
Source: eNeuro. 2025 Jan 28;12(1):ENEURO.0417-24.2024. doi: 10.1523/ENEURO.0417-24.2024 (PMC11801231; doi:10.1523/ENEURO.0417-24.2024)
Supplement: Table 1-1 — Statistical analyses. Download Table 1-1, DOCX file. [file eneuro-12-ENEURO.0417-24.2024-s001.docx]

**Supplementary Table 1. Statistical analyses.**

| **Figure** | **Test Name** | **Assumptions** | **Test Statistics** | **Test *p* Value** | **Multiple Comparisons** | ***p* Value** |
| --- | --- | --- | --- | --- | --- | --- |
| Fig 1F | One-way RM ANOVA | Homoscedasticity plot assessed and sphericity not assumed, Geisser-Greenhouse Epsilon = 0.5004, Residual plot approximately normal, QQ plot assessed and data is approximately normal, Holms-Sidak multiple comparisons test | F(2.002,240.3) = 120.6 | *p* < 0.0001 | PreTone vs. Tone 1  Shock 1 vs. Shock 2  Tone 1 vs. Tone 2  Tone 1 vs. Shock 1  Tone 2 vs. Shock 2  Shock 1 vs. Tone 2 | *p* = 0.2411  *p* < 0.0001  *p* = 0.0097  *p* < 0.0001  *p* < 0.0001  *p* < 0.0001 |
| Fig 1G | Spearman’s correlation | QQ plot assessed and data is non-normal, Did not pass Shapiro-Wilk test for normal distribution, Selection of spearman instead of Pearson correlation, Cells/speed from one mouse excluded due to improper save settings prior to acquisition of one behavior video | r = 0.4446  95% Confidence Interval 0.33-0.5414 | *p* < 0.001 |  |  |
| Fig 1H | Paired t-test | QQ plot approximately normal, Residual plot approximately normal, variances significantly different | t_8_ = 4.368 | *p* = 0.0014 |  |  |
| Fig 1J | Welch’s unpaired t-test | QQ plot approximately normal, residual plot approximately normal, Variances significantly different | T_145_ = 6.543 | *p* = 0.0001 |  |  |
| Fig 1K | Paired t-test | QQ plot approximately normal, residual plot approximately normal | t_89_ = 1.998 | *p* = 0.0488 |  |  |
| Fig 1M | Paired t-test | QQ plot approximately normal, residual plot approximately normal, cells from one mouse excluded due to improper save settings prior to acquisition on one behavior video | t_82_ = 2.423 | *p* = 0.0176 |  |  |
| Fig 2G | Paired t-test,  *n* = 7 | Variances not significantly different, Residual and QQ plots were approximately normal | t_6_ = 5.081 | *p* = 0.0023 |  |  |
| Fig 2I | Two-way RM ANOVA: Distance,  *n* = 5 ChR2  *n* = 11 YFP | Sphericity not assumed, matching is effective, 173987 and 173231 excluded for jump from apparatus | Stim x Transgene: F(5,70) = 1.907  Stim: F(3.594,50.31) = 14.19  Transgene: F(1,14) = 1.950  Subject: F(14,70) = 9.117 | *p* = 0.1041  *p* < 0.0001  *p* = 0.1844  *p* < 0.0001 | Within groups  Between groups | ns  ns |
| Fig 2J | Two-way RM ANOVA: Jumps,  *n* = 7 ChR2  *n* = 11 YFP | Sphericity not assumed, matching is effective, | Stim x Transgene: F(5,80) = 8.526  Stim: F(1.376, 22.02) = 8.526  Transgene: F(1,16) = 8.517  Subject: F(16,80)= 3.337 | *p* < 0.0001  *p* = 0.0043  *p* = 0.0100  *p* = 0.0002 | Within groups  Between groups | ns  ns |
| Fig 2K | Two-way RM ANOVA: Time Immobile,  *n* = 5 ChR2  *n* = 11 YFP | Sphericity not assumed, matching is effective, 173987 and 173231 excluded for software tracking error, exclude first off bin | Stim: F(3.1,43.39) = 2.861  Stim x Transgene: F(1,16) = 4.792  Transgene: F(4,56) = 1.388 | *p* = 0.0312  *p* = 0.1415  *p* = 0.1513 | Within groups  Between groups | ns  ns |

| Fig 2L | Two-way RM ANOVA: Grooming,  *n* = 7 ChR2  *n* = 11 YFP | Sphericity not assumed, matching is effective, Sidak correction for multiple comparisons | Stim x Transgene: F(5,80) = 5.027  Stim: F(2.509,40.14) = 7.549  Transgene: F(1,16) = 0.6117  Subject: F(16,80) = 5.188 | *p* = 0.0005  *p* = 0.0008  *p* = 0.4456  *p* < 0.0001 | Within groups  ChR2  Off1 vs. Off 2  On1 vs OfF1  All others  YFP  All  Between groups  Off 2  All others | *p* = 0.0097  *p* = 0.0213  ns  ns  *p* = 0.0253  ns |
| --- | --- | --- | --- | --- | --- | --- |
| Fig 2M | Two-way RM ANOVA: Rearing,  *n* = 7 ChR2  *n* = 11 YFP | Sphericity not assumed, matching is effective, Sidak correction for multiple comparisons | Stim x Transgene: F(5,80) = 2.648  Stim: F(2.858,45.73) = 2.165  Transgene: F(1,16) = 10.97  Subject: F(16,80) = 3.976 | *p* = 0.0288  *p* = 0.1079  *p* = 0.0044  *p* < 0.0001 | Within groups  Between groups  Off 3  On 3 | ns  *p* = 0.0188  *p* = 0.0344 |
| Fig 2N | Two-way RM ANOVA: Time in center,  *n* = 5 ChR2  *n* = 11 YFP | Sphericity not assumed, matching is effective, 173987 and 173231 excluded for software tracking error, exclude first off bin | Stim x Transgene: F(5,70) = 2.409  Stim: F(3.260,45.63) = 1.433  Transgene: F(1,14) = 0.1650  Subject (14,70) = 6.437 | *p* = 0.0449  *p* = 0.2439  *p* = 0.6907  *p* < 0.001 | Within groups  Between groups | ns  ns |
| Fig 3B | Welch’s t-test: Time in ON-zone, *n* = 7 ChR2  *n* = 11 YFP | F test to compare variance (p = 0.0971) | t_16_ = 13.12 | *p* < 0.0001 |  |  |
| Fig 3C | Welch’s t-test: Avg duration of visit to ON-zone,  *n* = 7 ChR2  *n* = 11 YFP | F test to compare variance (p = 0.0323) | t_16_ = 9.387 | *p* < 0.0001 |  |  |
| Fig 3D | Welch’s t-test: Entries to ON-zone,  *n* = 7 ChR2  *n* = 11 YFP | F test to compare variance (p = 0.0677) | t_16_ = 1.464 | *p* = 0.1627 |  |  |
| Fig 3E | Welch’s t-test: Time immobile,  *n* = 7 ChR2  *n* = 11 YFP | F test to compare variance (p = 0.0572) | t_16_ = 6.735 | *p* < 0.0001 |  |  |
| Fig 3F | Two-way ANOVA with Sidak multiple comparison test,  *n* = 7 ChR2  *n* = 11 YFP | Sphericity not assumed, QQ plot shows data approximately normal | Zone x Transgene: F(1,16) = 31.98  Zone: F(1,16) = 45.17  Transgene: F(1,16) = 21.06  Mouse F(16,16) = 0.9642 | *p* < 0.0001  *p* < 0.0001  *p* = 0.0003  *p* = 0.5286 | Between groups  On  Off  Within groups  ChR2  YFP | *p* = 0.6619  *p* < 0.0001  *p* < 0.0001  *p* = 0.6465 |
| Fig 3G | Two-way ANOVA with Sidak multiple comparison test,  *n* = 7 ChR2  *n* = 11 YFP | Sphericity not assumed, QQ plot shows data approximately normal | Zone x Transgene: F(1,16) = 3.495  Zone: F(1,16) = 0.0037  Transgene: F(1,16) = 26.17  Mouse (16,16) = 4.084 | *p* = 0.0800  *p* = 0.9524  *p* = 0.0001  *p* = 0.0038 | Between groups  On  Off  Within groups  ChR2  YFP | *p* < 0.0001  *p* = 0.0014  *p* = 0.4146  *p* = 0.3050 |
| Fig 3H | Two-way ANOVA with Sidak multiple comparison test,  *n* = 7 ChR2  *n* = 11 YFP | Sphericity not assumed, QQ plot shows data approximately normal | Zone x Transgene: F(1,16) = 0.8011  Zone: F(1,16) = 7.138  Transgene: F(1,16) = 17.43  Mouse F(16,16) = 2.184 | *p* < 0.3840  *p* = 0.0167  *p* = 0.0007  *p* = 0.0644 | Between groups  On  Off  Within groups  ChR2  YFP | *p* = 0.0116  *p* = 0.0008  *p* = 0.4708  *p* = 0.0226 |
| Fig 4 | Three-way RM ANOVA, *n* = 5 ChR2  *n* = 10 YFP |  | Day: F(2,82) = 1.838  Transgene: F(1,82) = 5.517  Phase: F(1,82) = 48.27  Day x Transgene: F(2,82) = 1.377  Day x Phase: F(2,82) = 0.6065  Transgene x Phase: F(1,82) = 14.59  Day x Transgene x Phase: F(2,82) = 0.303 | *p* = 0.1656  *p* = 0.212  *p* < 0.0001  *p* = .2582  *p* = 0.5477  *p* = 0.0003  *p* = 0.7388 | Within groups  ChR2  Day 1 In vs Post  Day 2 In vs Post  Day 3 In vs Post  YFP  Day 1 In vs Post  Day 2 In vs Post  Day 3 In vs Post  Between groups  Day 1 In  Day 2 In  Day 3 In  Day 1 Post  Day 2 Post  Day 3 Post | *p* = 0.9914  *p* = 0.0582  *p* = 0.9888  *p* = 0.9979  *p* > 0.9999  *p* = 0.9974  *p* = 0.9989  *p* = 0.9897  *p* = 0.9979  *p* = 0.0110  *p* < 0.0001  *p* < 0.0060 |
| Fig 5A | T-test with probabilistic Threshold-free Cluster Enhancement (pTFCA) |  | Heatmap for t scale on figure | *p* < 0.05 marked |  |  |
